# A retrospective observational study of length of stay in hospital after colorectal cancer surgery in England (1998–2010)

**DOI:** 10.1097/MD.0000000000005064

**Published:** 2016-11-28

**Authors:** Ariadni Aravani, Elizabeth F. Samy, James D. Thomas, Phil Quirke, Eva J.A. Morris, Paul J. Finan

**Affiliations:** a Cancer Epidemiology Group, Section of Epidemiology and Biostatistics, Leeds Institute of Cancer & Pathology, University of Leeds; b Knowledge and Intelligence Team (Northern & Yorkshire); c National Cancer Registration Service (Northern & Yorkshire), Public Health England; d Leeds Institute of Cancer & Pathology, University of Leeds, St James's University Hospital, Leeds, UK.

**Keywords:** colorectal cancer, length of stay, major resection

## Abstract

The National Health Service (NHS) is facing financial constraints and thus there is considerable interest in ensuring the shortest but optimal hospital stays possible. The aim of this study was to investigate patterns of postoperative length of stay (LOS) stay across the English NHS and to identify factors that significantly influence both optimal and prolonged LOS.

Data were obtained from the National Cancer Data Repository (NCDR). National patterns of LOS were examined and multilevel mixed effects logistic regression was used to study factors associated with an “ideal” (≤5 days) or a prolonged (≥21 days) LOS in hospital after major resection. Funnel plots were used to examine variation across hospitals in both risk-adjusted and unadjusted LOS.

All 240,873 individuals who underwent major resection for colorectal cancer were diagnosed between 1998 and 2010 in the English NHS. The overall median LOS was 10 (interquartile range [IQR] 7–14 days) days, but it fell over time from 11 (IQR 9–15) days in 1998 to 7 (IQR 5–12) days in 2010. The proportion of people experiencing “ideal” LOS increased dramatically from 4.9% in 1998 to 34.2% in 2010, but the decrease in the proportion of patients who experienced a prolonged LOS was less marked falling from 11.2% to 8.4%, respectively. Control charts showed that there was significant variation in short and prolonged LOS across NHS trusts even after adjustment for case-mix.

Significant variation in LOS existed between NHS hospitals in England throughout period 1998 to 2010. Understanding the underlying causes of this variation between surgical providers will make it possible to identify and spread best practice, improve services, and ultimately reduce LOS following colorectal cancer surgery.

## 1 Introduction

Colorectal cancer is a common disease in the UK with over 41,000 new cases diagnosed each year.^[1]^ The cost of diagnosing and treating these patients is considerable,^[2]^ and as the National Health Service (NHS) is facing significant funding constraints,^[3]^ organizing services to be both high-quality and cost-effective is important. Colorectal cancer is predominantly treated surgically and the major expense in managing the disease is associated with resection of the tumour and the associated postoperative length of stay (LOS).^[2]^ There is considerable interest, therefore, in ensuring the shortest hospital stays and the NHS has invested, among other initiatives, in minimally invasive surgery and enhanced recovery programmes to optimize surgical pathways.^[4]^

The length of time an individual spends in hospital following their surgery has been suggested as a marker of the quality of care. The accepted “ideal” LOS resulting from optimal surgery and enhanced recovery programmes is deemed to be 5 days or fewer. The proportion of individuals with hospital stays at or below this threshold has been routinely used, therefore, as a measure of good practice.^[5]^ Conversely, excessive or prolonged LOS has also been suggested as an indicator of the effectiveness of care. Overall, however, there is significant interest in monitoring hospital stays and publishing comparative analyses across the different multidisciplinary teams (MDTs) providing care in an effort to improve standards. Reliably identifying institutions with “outlying” practice is, however, difficult for several reasons.^[6]^

First, numerous factors may influence postoperative LOS including the age of the patient, the presence of concomitant disease, the site and stage of the tumour and the method of presentation, type of operation performed, and whether laparoscopic or not.^[7–11]^ Different MDTs treat populations with very different characteristics and so robust comparison demands the differing balance of these factors to be incorporated in “risk-adjusted” analyses. Furthermore, the annual number of patients operated for colorectal cancer differs between hospitals. Greater variability in LOS may be observed among hospitals with lower workloads compared to those with higher workloads simply by chance. Thus, appropriate adjustment for workload must be undertaken to reliably compare providers. Finally, national comparisons demand national population-based data containing information on factors likely to influence LOS. These have only relatively recently become available in the National Cancer Data Repository (NCDR).^[12,13]^ This is a resource that includes linked cancer registry (CR) and Hospital Episode Statistics (HES) data. This study aims, therefore, to make use of these data and apply robust methods to investigate patterns of postoperative LOS stay across the English NHS. It seeks to identify factors that significantly influence both optimal and prolonged LOS and make risk-adjusted comparisons between all providers treating this disease, with the ultimate purpose of feeding these information back to Trusts to enable them to do “root cause analyses” of LOS.

## 2 Methods

All individuals diagnosed with a first primary colorectal cancer (*International Classification of Diseases Version 10* [*ICD-10*] C18-C20)^[14]^ between 1998 and 2010, and who had undergone a major resection^[15]^ for their disease in an NHS hospital, were identified, in the NCDR. Information on date of diagnosis, age, sex, deprivation (measured via the income domain of the Index of Multiple Deprivation [IMD] 2004), site of tumour, and Dukes’ stage were extracted from the cancer registry dataset. Information on the type of surgery, date of surgery, and allocation of patients into Trusts were derived, using standard algorithms,^[12,15]^ from the HES component of the NCDR.

A Charlson comorbidity index^[16]^ was derived for each individual in the cohort, taking into account diagnoses (excluding cancer) from any hospital admissions in the year before the diagnosis of colorectal cancer. The cancer component of the Charlson index was derived from the cancer registry information found in NCDR and the score for any other cancers in the year before colorectal cancer was added to the score obtained from HES data. The Charlson score was categorized as: 0, 1, 2, and ≥3 with higher scores indicating greater comorbidity.

LOS was defined as the time from the date of major resection to the date of discharge or death, whichever came first. Individuals with a negative LOS (due to errors in dates recorded in the routine data) were excluded. Individuals experiencing the optimal stay of ≤5 (including the day of discharge but excluding those who died within this time) were identified as were those with a prolonged LOS ≥90^th^ percentile (21 days). The overall median LOS and the proportion of individuals in both the optimal and prolonged LOS categories were then calculated by the patient and management characteristics of age, year of diagnosis, sex, IMD category, tumour site, admission method, Charlson comorbidity score, Dukes’ stage of disease at diagnosis, and operation type (emergency/elective). Variation in LOS in relation to the approach to surgery (open, laparoscopic or laparoscopic conversion) was also investigated but as laparoscopic procedures were not consistently coded in HES before 2006 these analyses were limited to cases diagnosed after this time. Mann–Whitney, *χ*^2^, or *t* tests were used to assess the statistical significance of any differences in LOS.

Dukes’ stage of disease is an important prognostic factor in colorectal cancer, but information was missing for this factor from 12% of cases. Analysis based on complete data only would restrict the assessment of LOS to 88% of the patients introducing a risk of bias and loss of information. These missing data were imputed,^[17]^ therefore, using the ICE command in Stata (Version 13.1), an iterative multivariable regression technique, using ordered logistic regression with 5 imputations and 10 cycles of regression switching. Dukes’ stage data were assumed to be “missing at random” (MAR), which means that given the observed data, the missingness mechanism does not depend on the unseen data. The MAR assumption is possible because all the variables included in the analyses or potentially predictive of Dukes’ stage missing values were included in the imputation model.

The imputation model consisted of variables: Length of hospital stay, age at diagnosis, sex, workload of the trust, Dukes’ stage, IMD income category, tumour site, year of diagnosis, year of major resection, admission method (elective or emergency), Charlson comorbidity score, hospital of management, and cancer registry region. The models used to assess prolonged LOS were applied to both the complete dataset and the imputed dataset for sensitivity analysis purposes.

Two models were used to identify factors associated with both the ideal and prolonged LOS. These were multilevel mixed effects logistic regression with a hierarchy of patients (level 1) nested within hospitals (level 2). The response variable was either the ideal or prolonged LOS. Explanatory variables in the risk-adjusted model were age at diagnosis, sex, IMD income category, tumour site, year of diagnosis, Charlson comorbidity score, Dukes’ stage at diagnosis, type of operation (elective or emergency), and approach to surgery (open, laparoscopic, and converted). The results are presented as an odds ratio (OR) with 95% confidence interval (CI) for each explanatory variable. OR is the ratio of the odds of the event of interest (i.e., postoperative LOS <5 days) in one group (i.e., females) over the odds of the event of interest in the baseline group (i.e., males).

Funnel plots were used to compare both ideal and prolonged LOS rates between trusts, using the Spiegelhalter approach.^[18]^ Ideal and prolonged LOS ratios were calculated for each MDT from each individual's probability of staying in hospital in the defined periods, derived from models based on the imputed dataset. Ideal and prolonged LOS rates were then calculated by multiplying the MDT-specific ratios by the average national rates (shown as a horizontal line on the funnel plots). These MDT-specific ratios were then plotted against MDT surgical workload (number of major resections in the study period) using the “funnelcompar” command in Stata with 95% (inner line) and 99.8% (outer line) control limits. Laney's approach^[19]^ was used to deal with overdispersion. MDTs with rates outside the 99.8% control limits were considered to be “outliers.”

Ethical approval for this study was obtained from East of Scotland Research Ethics Committee Reference (No. 08/S0501/66).

## 3 Results

A total of 243,558 individuals were identified as having undergone a major resection for a first primary bowel cancer diagnosed between 1998 and 2010 in one of 150 English MDTs. Of these, 2685 had dates of discharge that were before their date of major resection giving a negative LOS. These individuals were, therefore, excluded leaving a full study population of 240,873. As information on the approach to surgery was only available from 2006 onwards the study population for analyses investigating the use of laparoscopy was limited to the eligible 99 100 diagnosed and managed post that date.

**Figure 1:**
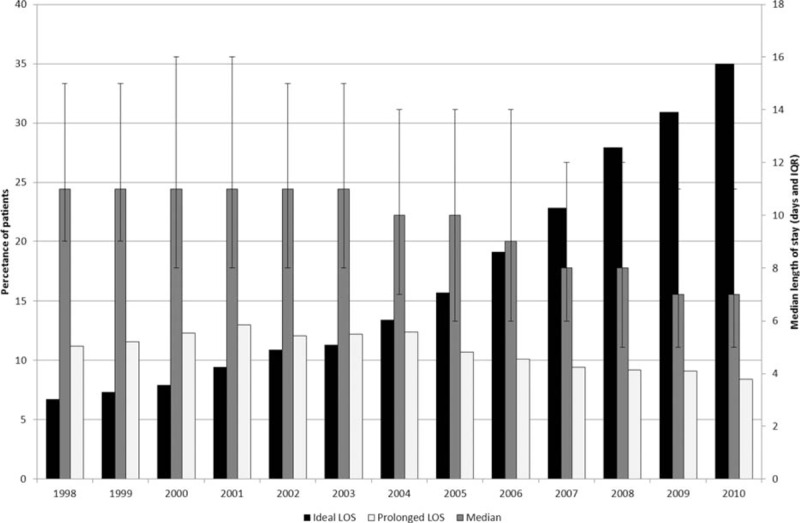
Changes in median, ideal, and prolonged length of stay over time.

The overall median LOS for the 1998 to 2010 and 2006 to 2010 study periods were 10 (interquartile range [IQR] 7–14 days) and 8 (IQR 5–13) days, respectively (Fig. 1), but over time there were significant changes in practice. Although the overall median LOS decreased gradually falling from 11 (IQR 9–15) days in 1998 to 7 (IQR 5–12) days in 2010 the proportion of the population who experienced a short “ideal” LOS increased dramatically from 4.9% in 1998 to 34.2% in 2010 (Table 1 and Fig. 1). The reduction in the proportion of patients who experienced a prolonged LOS was much less marked falling from 11.2% to 8.4% over the same period. LOS also varied considerably in relation to the characteristics of the population (Table 1). Overall median LOS increased with increasing age (12 days [IQR 7–18] for those >80 years vs. 8 days [IQR (6–12] for those aged ≤50) and socioeconomic deprivation (11 days [IQR 7–16] for those living in the most deprived areas versus 9 days (IQR 7–14) for those in the most affluent areas) and was longer for those with rectal cancer (11 days [IQR 8–16]) compared to colon cancer (9 days [IQR 6–14]).

**Table 1:**
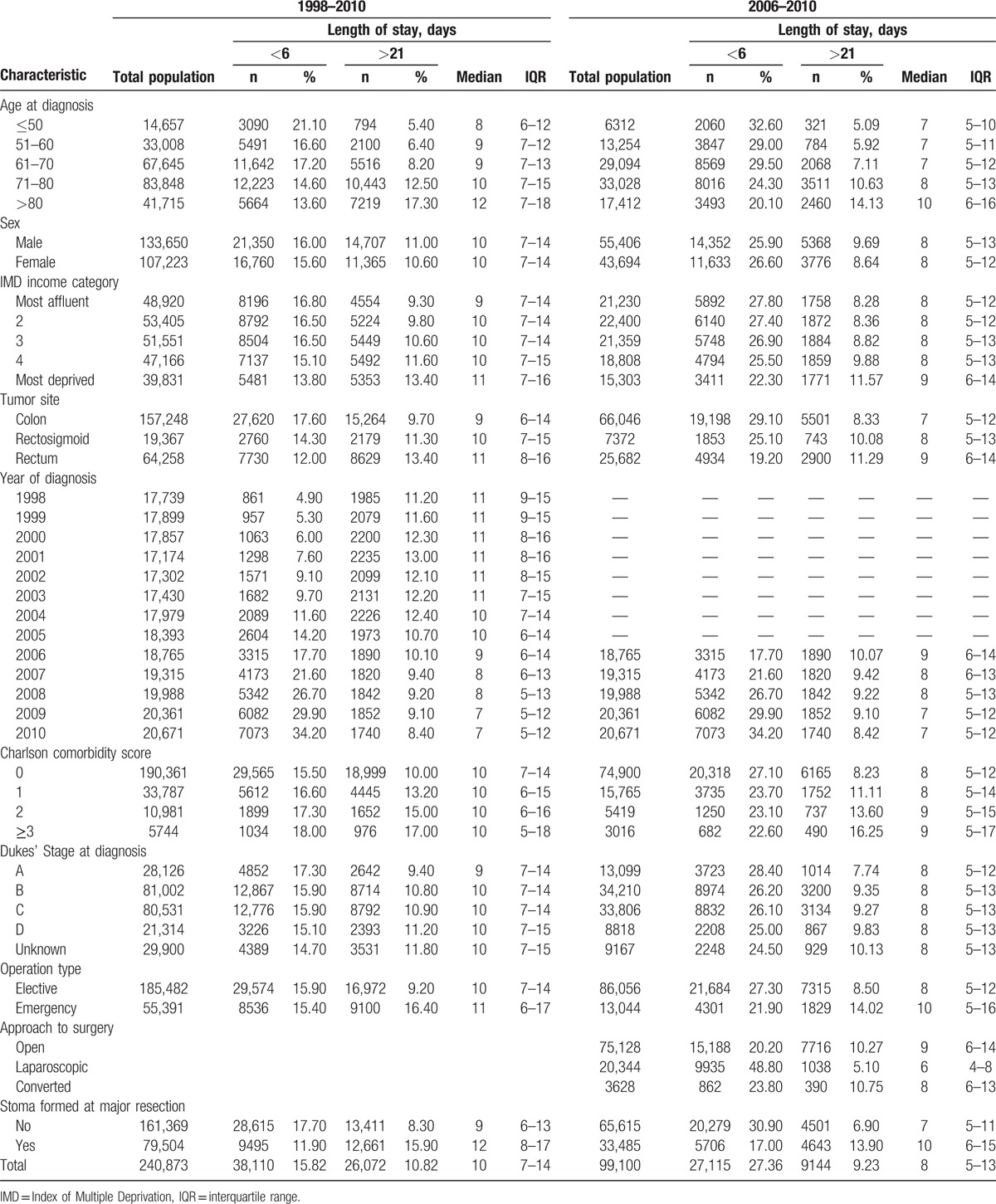
Characteristics of the study population in relation to length of stay.

In the 1998 to 2010 analysis, the proportion of people experiencing an “ideal” LOS was greatest in those in younger age (21.1% in those ≤50 vs. 13.6% in those >80, *P* < 0.001), the more socioeconomically affluent (16.8% in the most affluent group versus 13.8% in the most deprived, *P* < 0.001), in those with colon cancer rather than rectal cancer (17.6% vs. 12.0%, *P* < 0.001), in those with early stage disease (17.3% in Dukes A patients versus 15.1% in stage D, *P* < 0.001) and in those who did not have a stoma formed at major resection (17.7% vs. 11.9%, *P* < 0.001). The formation of stoma is associated with the cancer site, with 69% of patients diagnosed with rectal cancer getting a stoma in contrast to the 31% of those who were diagnosed with colon cancer. The relationship with comorbidity was complex. In the 1998 to 2010 analysis, the proportion experiencing an optimal LOS increased with increasing Charlson comorbidity scores, but in the 2006 to 2010 analysis, the effect was reversed. This may be related to the improvements in comorbidity recording in routine data over time. There was no clinically significant difference in the proportion having an optimal stay in relation to operation type in the 1998 to 2010 analysis (15.9% for elective operation versus 15.4% for emergencies, *P* < 0.001 [statistically significant difference owing to large group sizes]), but in the 2006 to 2010 analysis, a significant greater proportion of elective patients experienced a short hospital stay (27.3% vs. 21.9%, *P* < 0.001).

In the 1998 to 2010 analysis, the proportion of people experiencing a prolonged LOS was greatest in those in older age groups (17.3% in those >80 compared to 5.4% in those ≤50, *P* < 0.001), the more socioeconomically deprived (13.4% in those living in the most deprived areas vs. 9.3% in the most affluent *P* < 0.001) and more comorbid groups (17% in Charlson score ≥3 vs. 10% in Charlson score 0, *P* < 0.001) and was also elevated in those who underwent emergency surgery (16.4% vs. 9.2%, *P* < 0.001). Prolonged LOS was also more common in individuals with rectal rather than colonic tumors (13.4% vs. 9.7%, *P* < 0.001) and in those who had a stoma formed at major resection (15.9% vs. 8.3%, *P* < 0.01).

The results of the model investigating the odds of an optimal LOS for the period 2006 to 2010 are shown in Table 2. Factors associated with reduced odds of an optimal LOS included increasing age (OR 0.81 95% CI 0.79–0.82, *P* < 0.001), increasing socioeconomic deprivation (OR 0.81 95% CI 0.77–0.86, *P* < 0.001 for the most deprived category versus the most affluent), having a rectal tumour (OR 0.49 95% CI 0.47–0.51, *P* < 0.001 compared to a colon tumour), being admitted as an emergency (OR 0.88 95% CI 0.84–0.91, *P* < 0.001 for emergency procedures versus elective), higher levels of comorbidity (OR 0.87 95% CI 0.79–0.95, *P* < 0.001 for Charlson score ≥3 vs. 0), with increasing stage at disease (OR 0.91 95% CI 0.85–0.98, *P* < 0.001 Dukes D compared to Dukes A). In contrast, the odds of an optimal LOS increased significantly over time (OR 1.17 [per year] 95% CI 1.16–1.19, *P* < 0.001) and in those undergoing laparoscopic surgery (OR 3.58 95% CI 3.45–3.72, *P* < 0.001 compared to open surgery). There was no statistically significant difference in the odds of having an ideal LOS in those whose laparoscopic surgery was converted to open surgery (OR 1.06 95% CI 0.97–1.15, *P* = 0.185).

**Table 2:**
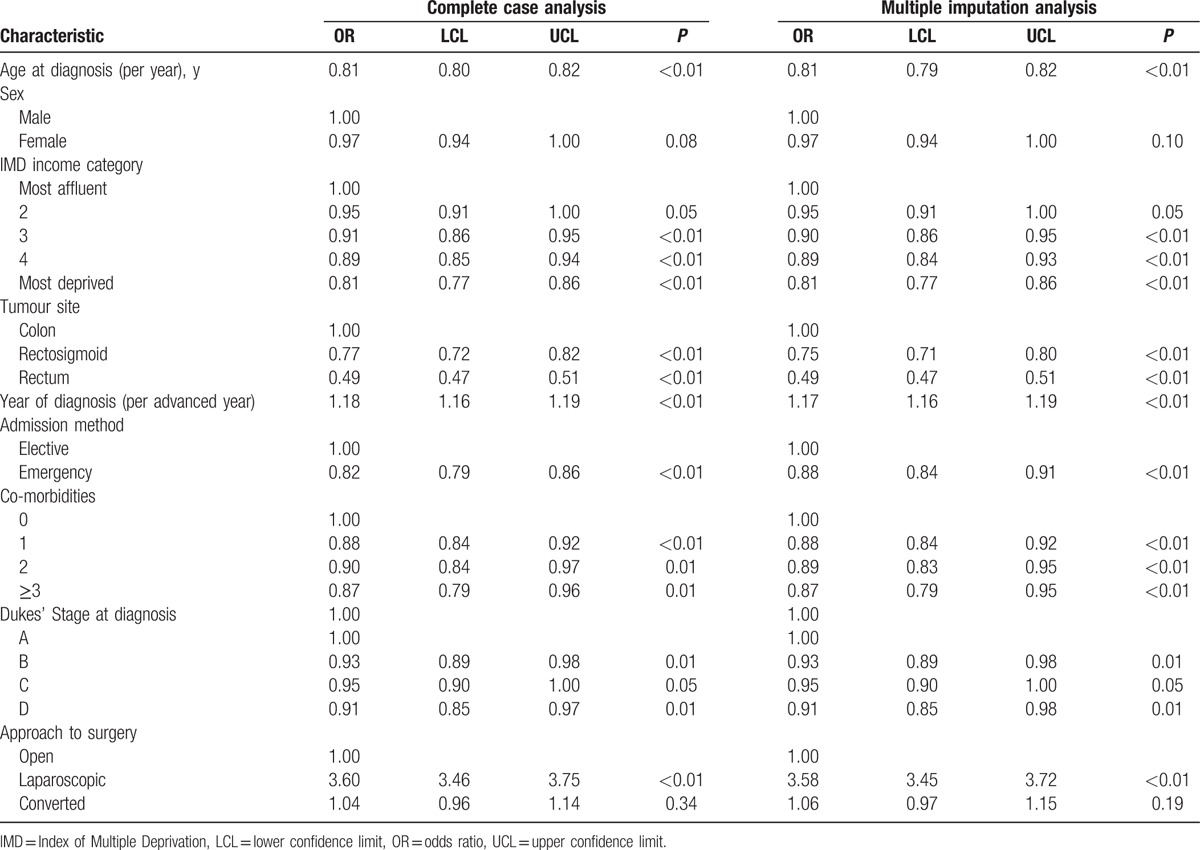
Multivariable analysis investigating the odds of an ideal length of stay (2006–2010).

**Figure 2:**
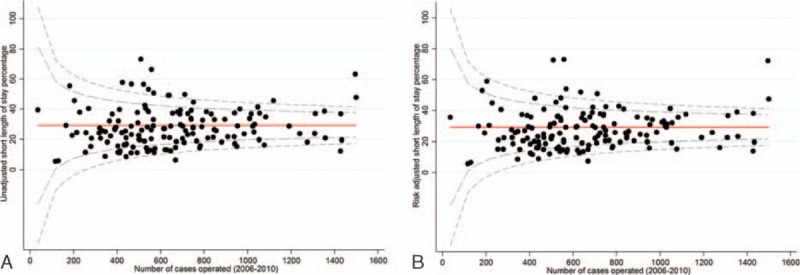
(A) Risk-unadjusted short length of stay by NHS trust for patients with colorectal cancer who underwent a major resection: England, patients diagnosed between 2006 and 2010. (B) Risk-adjusted short length of stay (adjusted for age, sex, deprivation, tumour site, year of diagnosis, admission method, Charlson comorbitidy score, Dukes’ stage, and approach to surgery).

This model was used to generate risk-unadjusted and risk-adjusted funnel plots investigating the proportion of patients experiencing an optimal LOS across all the English colorectal MDTs managing these patients (Fig. 2A and B). Thirteen MDTs had significantly higher risk-unadjusted rates and 7 MDTs had significantly higher risk-adjusted rates of ideal LOS than was expected (i.e., outside the upper 99.8% control limit). The equivalent figures for the risk-unadjusted and adjusted analyses for lower rates than expected were 2 and 4 MDTs, respectively.

Conversely, the results of the model investigating the odds of a prolonged LOS for period 2006 to 2010 are shown in Table 3. The odds of a prolonged LOS were statistically significantly greater in the elderly (OR 1.14 95% CI 1.38–1.44, *P* < 0.001 per year increase in age), in those residing in more socioeconomically deprived areas (OR 1.28 95% CI 1.19–1.39, *P* < 0.001 for those most deprived category compared to the most affluent), in those with rectal tumors (OR 2.04 95% CI 1.93–2.15, *P* < 0.001 for rectal tumors compared to colon), in those presenting as an emergency (OR 2.10 95% CI 1.99–2.22, *P* < 0.001), in those with greater comorbidity (OR 1.66 95% CI 1.50–1.85, *P* < 0.001 for Charlson score ≥3 vs. 0) and with increasing stage at disease (OR 1.13 95% CI 1.01–1.27, *P* = 0.025 for Dukes D vs. Dukes A). The odds of a prolonged LOS was significantly reduced in those undergoing laparoscopic surgery (OR 0.55, 95% CI 0.51–0.59, *P* < 0.001) but was increased in those who underwent laparoscopic surgery that was converted to open surgery (OR 1.19, 95% CI 1.06–1.33, *P* < 0.001).

**Table 3:**
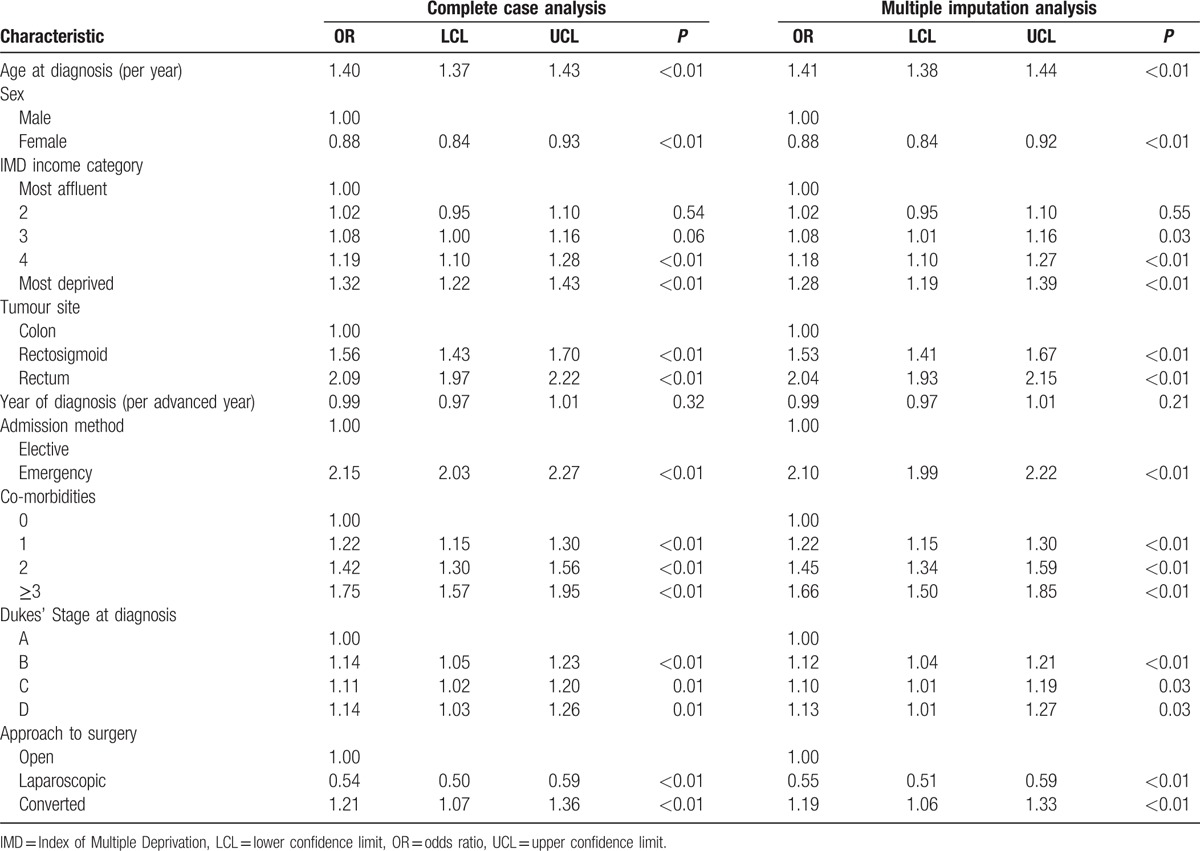
Multivariable analysis investigating the odds of a prolonged length of stay (2006–2010).

**Figure 3:**
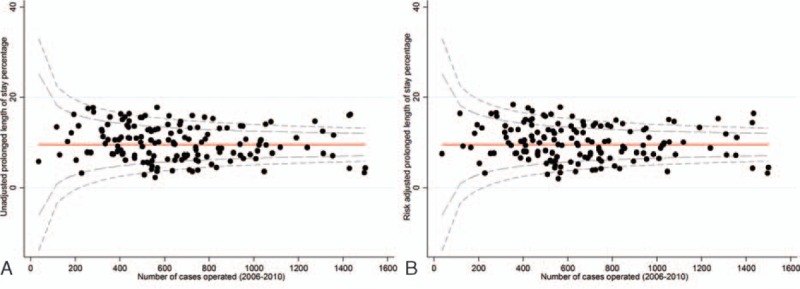
(A) Risk-unadjusted prolonged length of stay by NHS trust for patients with colorectal cancer who underwent a major resection: England, patients diagnosed between 2006 and 2010. (B) Risk-adjusted prolonged length of stay (adjusted for age, sex, deprivation, tumour site, year of diagnosis, admission method, Charlson comorbitidy score, Dukes’ stage, approach to surgery).

Twelve MDTs had a statistically significantly greater proportion of patients experiencing a prolonged LOS than would be expected in the risk-adjusted and unadjusted analyses respectively (Fig. 3A and B). In contrast, 12 and 11 MDTs had a statistically significantly lower rate of patients experiencing a prolonged LOS than was expected in the risk-adjusted and -unadjusted analyses, respectively.

## 4 Discussion

This retrospective population-based study comprehensively quantifies patterns of postoperative LOS following major colorectal cancer surgery across the English NHS. The study shows that over a 13-year period, the overall median LOS has statistically significantly reduced. This is in large part because of a major increase in the proportion of people experiencing an optimal LOS of ≤5 days, which was promoted as a measure of good care after the introduction of enhanced recovery programmes.^[5,20–23]^ Conversely, there was a much less marked reduction in the proportion of people experiencing a prolonged LOS of ≥21 days. In addition, there was significant variation across the population with LOS proving longer in the elderly, those living in socioeconomically deprived areas, with rectal cancers, with greater comorbid disease and in those admitted as an emergency. LOS was statistically significantly reduced in those whose surgery was successfully undertaken laparoscopically. Statistically significant variation, independent of case mix was also observed between the colorectal MDTs. Given that the average cost per bed-day for a person undergoing colorectal cancer surgery is £441, according to the MRC Conventional versus Laparoscopic-Assisted Surgery in Colorectal Cancer trial (CLASSIC),^[24]^ a median LOS reduction from 11 days in 1998 to 7 days in 2010 saved the NHS an estimated £1 764 per patient (around £32 M per year on average).

The overall median LOS is broadly comparable to that shown in other UK studies. For example, the National Bowel Cancer Audit Programme, which captures data submitted voluntarily by the majority of hospitals in the UK, reported a comparable median LOS of seven and nine days for colon and rectal cancer patients, respectively.^[5]^ Another study based on all elective malignant and nonmalignant colorectal surgeries across the same population did, however, report a longer median LOS.^[9]^ This is unexpected given that study was limited to elective surgery and included nonmalignant operations too. It is not clear why their findings are discrepant, but it may be a result of the use of total LOS rather than the definition used here of postoperative LOS. The relevant total LOS derived from the 2 studies are broadly comparable (results not shown). The LOS for English patients also appears to compare relatively favorably to equivalent international figures.^[8,10,25]^

The findings of this study support those previously published^[26–29]^ that indicate there are numerous characteristics that consistently influence LOS. For example, longer hospitals stays were associated with increasing patient age, comorbidity, socioeconomic deprivation, stage of disease, and following emergency operations. The risk of surgery increases with many of these factors and greater surgical challenge would logically predispose to greater recovery times. Furthermore, the longest stays were observed in those undergoing surgery for rectal cancer. This is a result of the fact that postoperative stays of individuals receiving stomas, which currently rectal cancers demand more frequently, are longer by a median of 3 days than those of people who are able to maintain the continuity of the bowel.

A potential limitation of the study is that the case-mix adjustment was inadequate because of the routine nature of the data analyzed. For example, the Charlson index was used as a measure of comorbidity. Although this index is able to robustly predict prognosis and, consequently, is used routinely to assess the influence of comorbidity in many population-level analyses, it is a relatively crude measure, as it only incorporates information on illness that necessitated a hospital stay in the year before diagnosis. Individuals may possess other comorbidities that still have an important influence on surgical outcomes but the routine data available in the NCDR does not yet allow these to be reliably identified and taken into consideration. In addition, the quality of coding in routine hospital data has improved over time leading to changes in the comorbidity information the Charlson Index relies on. (This may explain the unusual and opposing relationship of Charlson score and the proportion of people experiencing an ideal LOS in the 1998–2010 and the 2006–2010 analyses.) Likewise, a person's socioeconomic status should not influence the rate at which they recover from an operation but those living in more deprived areas are perhaps more likely to have unhealthy lifestyles and so pose a greater surgical risk and require a longer recuperation time or have social reasons for delayed discharge. Again the availability of more detailed case-mix information could significantly improve the strength of the results.

Another potential limitation of the study is its failure to take into account other outcomes related to LOS. For example, a rapid discharge may be inappropriate if the patient has not sufficiently recovered and, consequently, is rapidly re-admitted. Likewise, rapid postoperative deaths will positively influence LOS, but are a negative outcome. The methodology was adapted to ensure those who died rapidly postsurgery were excluded from the optimal LOS category, but a high number of postoperative deaths will, nevertheless, still reduce an MDT's overall LOS and this should be considered in any hospital level comparative analyses. No changes were made to the methodology to account for re-admission rates, but the routine data this study is based on would allow for this to be quantified and future analyses are, therefore, proposed in this area.

In an effort to increase the efficiency of services the NHS has, in recent years, made reducing LOS a priority. This study suggests that the numerous interventions adopted to reduce LOS, including the roll out of laparoscopic surgery^[30]^ and enhanced recovery programmes^[4]^ have had a significant impact. Although these interventions rapidly rolled out between 2006 and 2010 there is not an accurate way of recording whether or not the hospital has applied the concept of enhanced recovery. Overall median LOS has dropped and the proportion of patients experiencing an optimal stay has increased significantly. There has, however, been a much less marked reduction in the proportion of individuals experiencing a prolonged LOS. This may suggest that although initiatives to reduce LOS are effective, there remains a stubborn population of high-risk patients whose postoperative recovery times are likely to be substantial. Further quantification of the characteristics of this population would provide useful evidence to help develop future management strategies that could reduce the LOS for this group, such as screening or increase of colorectal cancer resections. However, with the aging population, it could also be anticipated that the proportion of individuals with comorbidity and other negative prognostic factors will increase resulting in a tendency for average LOS to increase. The optimization of diagnostic and treatment strategies will be required to meet this challenge and ensure an optimal postoperative LOS.
